# Neurodegeneration in Multiple Sclerosis: Symptoms of Silent Progression, Biomarkers and Neuroprotective Therapy—Kynurenines Are Important Players

**DOI:** 10.3390/molecules26113423

**Published:** 2021-06-05

**Authors:** Dániel Sandi, Zsanett Fricska-Nagy, Krisztina Bencsik, László Vécsei

**Affiliations:** 1 Albert Szent-Györgyi Clinical Centre, Department of Neurology, Faculty of General Medicine, University of Szeged, H-6725 Szeged, Hungary; sandi.daniel@med.u-szeged.hu (D.S.); fricska-nagy.zsanett@med.u-szeged.hu (Z.F.-N.); bencsik.krisztina@med.u-szeged.hu (K.B.); 2 MTA-SZTE Neuroscience Research Group, University of Szeged, H-6725 Szeged, Hungary; 3 Interdisciplinary Excellence Centre, University of Szeged, H-6725 Szeged, Hungary

**Keywords:** multiple sclerosis, kynurenine, neurodegeneration, biomarker, neuroprotection, therapy

## Abstract

Neurodegeneration is one of the driving forces behind the pathogenesis of multiple sclerosis (MS). Progression without activity, pathopsychological disturbances (cognitive impairment, depression, fatigue) and even optic neuropathy seems to be mainly routed in this mechanism. In this article, we aim to give a comprehensive review of the clinical aspects and symptomology, radiological and molecular markers and potential therapeutic targets of neurodegeneration in connection with MS. As the kynurenine pathway (KP) was evidenced to play an important role in the pathogenesis of other neurodegenerative conditions (even implied to have a causative role in some of these diseases) and more and more recent evidence suggest the same central role in the neurodegenerative processes of MS as well, we pay special attention to the KP. Metabolites of the pathway are researched as biomarkers of the disease and new, promising data arising from clinical evaluations show the possible therapeutic capability of KP metabolites as neuroprotective drugs in MS. Our conclusion is that the kynurenine pathway is a highly important route of research both for diagnostic and for therapeutic values and is expected to yield concrete results for everyday medicine in the future.

## 1. Introduction

**Figure 1:**
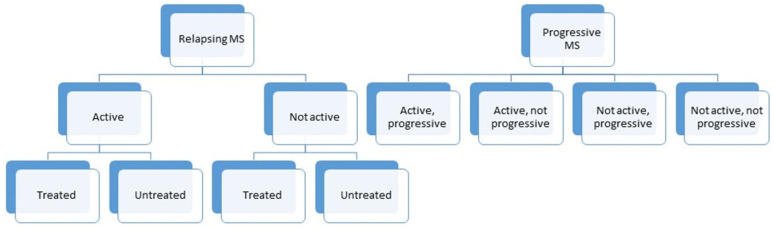
The new phenotypic classification of multiple sclerosis. MS, multiple sclerosis.

Multiple sclerosis (MS) is a chronic, demyelinating disease of the central nervous system (CNS) involving inflammation and progressive neurodegeneration. According to the new phenotypic classification published by Lublin in 2014, the core MS phenotypes are the relapsing–remitting disease and the progressive disease [1]. The relapsing course consists of the clinically isolated syndrome (CIS) and the “classical” relapsing–remitting (RRMS) clinical course. The primary progressive (PPMS), the secondary progressive (SPMS) and the progressive-relapsing phenotypes were categorized separately as progressive disease. In case of the latter, the patients can be grouped in four sub-groups: the active with progression (the patient has had a relapse and the level of disability is also gradually worsening), the active but without progression (the patient had relapses earlier), the inactive with progression (the patient has no relapses, but a confirmed worsening in expanded disability status scale (EDSS) score is detectable), and the stable form without disease activity and progression—as it can be seen in Figure 1.

In all clinical courses, disease activity has to be determined by clinical relapses and/or magnetic resonance imaging (MRI) findings, such as gadolinium-enhancing lesions or new/enlarging T2 lesions. In the case of progressive disease, 1-year-long progression, i.e., the continuous worsening of neurological dysfunction without full remission, has to be confirmed [2]. In general, MS starts between the age of 20–30 years, and after 10–15 years, it converts into progressive phase (SPMS) [3,4]. In some cases, the progression of disability starts from the beginning of the disease, with a higher age at onset (PPMS) [5].

Whatever the clinical course is, MS will result in the serious disability of the patient and not just a gradual worsening in their quality of life, but also in a severely elevated mortality risk if the disease is left untreated. Despite the great success of new therapies in the last two decades, MS is still an incurable condition and will remain so, until we understand the pathophysiological processes that drive the disease and can find reliable biomarkers to monitor its activity and progression. We have to focus on both of the basic mechanisms behind the disease: not just inflammation but neurodegeneration as well. In this review, after a short summary of the pathogenic mechanism currently revealed behind MS, we aim to give a comprehensive review of the processes in connection with the neurodegeneration of MS: silent clinical progression and symptomatology; the possible imaging and molecular biomarkers. We give special consideration to the metabolites of the kynurenine pathway. New evidence continuously shows its role in the pathology of MS and other neurodegenerative conditions, which were thought to be unrelated to MS, yet encompass similar symptomatology and neurodegenerative processes. Furthermore, kynurenine metabolites show promise in biomarker research and even as possible neuroprotective therapies in the future.

## 2. The Pathogenic Mechanism behind Multiple Sclerosis

It is generally believed that MS is a two-stage disease, first beginning with inflammation, which is followed by a neurodegenerative phase. However, increasing evidence from imaging and neuropathological assessments suggest that neurodegeneration is present from the very onset of the disease, and the switch from relapsing to progressive phase is the result of the decreased capacity of the compensatory mechanism of neuronal injury [6]. Pathogenetic examinations show differences between the relapsing and the progressive phases of MS in clinical signs, immunological processes and pathology. “Subtypes” of the progressive disease, primary and secondary progressive MS, however, differ in a quantitative and not in a qualitative manner. While the qualitative differences between the core phenotypes manifest in the presence of active white matter lesions and the level of inflammation in CNS, the quantitative differences between the progressive subtypes are not as clear [7]. Thus, what is the mechanism through which the pathologic processes damage the CNS? Additionally, if different, which processes dominate the different clinical phenotypes (and subtypes)? The answer is still not clear. However, we do know that one hallmark process is inflammation in MS, which is widely considered to be the initiator of the disease, and it can be categorized into two types.

The first involves lymphocytes entering the CNS across the damaged blood–brain barrier (BBB), recognizing the antigen and becoming activated and producing pro-inflammatory subjects [8]. It was believed previously that major histocompatibility complex (MHC) class II antigen restricted CD4+ T-lymphocytes play the most important role in this process, but recently, it was shown that at least in the early stage, when the lesions expand in the CNS, the CD8+ T-lymphocytes and CD20+ B-lymphocytes exert a more important effect. The role of CD8+ T-cells in the disease process is not fully understood. These cells have similar characteristics to tissue resident memory cells [9]. In the brain of an MS patient, these CD8+ T-cells can proliferate, become activated and propagate clonally, which leads to local antigen recognition [10,11]. Although this process is characteristic to classical active lesions, CD8+ cells seem to have a role in the progressive phase as tissue resident memory cells with local activation causing neurodegeneration. The role of B-cells in the process was also confirmed by the therapeutic effect of anti-CD20 treatment in MS. Studies show that these cells can have regulatory functions, which is connected to their stage of differentiation [12]. The acute inflammatory process started by T- and B-cells results in the primary demyelination and axonal injury caused by activated microglia and macrophages. This is the time of the appearance of variable active focal plaques in CNS [13].

The second process occurs in the connective tissue spaces of the CNS, such as the meninges and in the large periventricular Virchow–Robin spaces [14,15]. This process can cause the slow expansion of the focal white matter lesions, subpial cortical demyelination and diffuse damage of the normal-appearing white and gray matter [16]. The CD20+ B cells are located in inflammatory aggregates in these areas of CNS and can transform into plasma cells in the process of lesion maturation.

These two inflammatory processes occur in parallel both in the relapsing–remitting and the progressive form of the disease, however; the first, classical inflammatory process is dominant in the early stage and declines with age and disease duration [17,18]. The second, slow process of the enlargement of lesions in normal-appearing white and gray matter and neurodegeneration is more frequent in the progressive phase. The B- and T-cells have roles in both: causing tissue damage and regulating inflammatory processes. The tissue damage is caused by a soluble factor, but similarly to the antigen, it has not been detected yet.

Thus, as we can see, there are abundant data that MS is primarily an immunological condition, where the dysregulation and the pathological activation of the immune system initiates the cascade and neurodegeneration is a subsequent consequence of the autoaggressive inflammation. However, this view has been challenged recently. There is accumulating evidence that the other hallmark process, neurodegeneration, is not simply present from the very beginning of the disease, but might even be the initiator of the pathological cascade resulting in MS. This hypothesis suggests that oligodendrocyte apoptosis, axonal damage and subsequent microglial activation is the initial step in the evolution of lesions in the absence of autoreactive immune cells [19,20,21]. This leads to a secondary autoimmune response and demyelination, which in turn leads to oligodendrocyte apoptosis, creating a feedback loop. Serious dysfunction of the mitochondria also heavily contributes to the process, as it plays a central role in oligodendrocyte apoptosis and the halting of the differentiation of oligodendrocyte progenitor cells [22,23]. Additionally, it was linked to the loss of small-diameter axons and lesion progression, and astrocytic dysfunction [24,25].

Whichever process is the initial in the pathogenesis of MS, neurodegeneration is a profound driving mechanism throughout the course of the disease. Its role can be felt in action in all aspects of multiple sclerosis, be it clinical, imaging or molecular. In the next section, we elaborate on the clinical aspects of neurodegeneration in MS.

## 3. Progression Independent of Relapse (and MRI) Activity (PIRA and PIRMA)

Patients with the relapsing–remitting course of MS accumulate neurological deteriorations, and after a period of time the disease switches into a secondary progressive phase. Thanks to the broad treatment opportunities, several MS patients do not show disease activity (no relapse, no active lesions). Despite this fully desired outcome, several patients, even at the beginning of the relapsing phase of the disease, show a continuous progression/worsening of their state which is completely independent from relapse activity.

PIRA and PIRMA has recently become a focus of clinical evaluations. A pivotal study from 2020 by Kappos et al. observed pooled data from the intention-to-treat population of two identical, phase 3, multicenter, double-blind, double-dummy, parallel-group randomized clinical trials (OPERA I and II) assessing the effectivity of ocrelizumab as compared to interferon-*β* (IFN-*β*) treatment [26]. The outcome of the evaluation was the confirmed disability accumulation (CDA) in one of three measures: EDSS score; the timed 25-feet walk test (T25W), a measure of lower limb function, or the 9-hole peg (9HP) test, a measure of the upper limb function at 3 and 6 months in regard to their temporal association with clinical relapses and/or MRI progression. The study found that roughly 16–25% of both arms (significantly lower proportion on the ocrelizumab arm) had confirmed disability accumulation and more importantly, 80–90% of this was due to PIRA. Even more interesting is the fact that though EDSS contributed mostly to the CDA during relapses, the overall contribution of 9HP and T25W were much higher to PIRA. These are highly surprising results, as patients in these clinical studies were at the beginning of their disease with short disease duration and in the relapsing phase of their disease. Earlier studies, with less rigorous design—including long-term data from the Tysabri Observational Program—also implied that disability accumulation is largely independent from commonly measured parameters [27,28,29].

These data heavily challenge our understanding of the pathomechanism of MS, even the “raison d’etre” of the current phenotypic classification. First, it is hard evidence pointing to the higher relevance of progression, associated with neurodegeneration in the pathomechanism of MS as compared to the measures of (acute) inflammatory processes. Since the introduction of the “no evidence of disease activity” (NEDA) concept, however, this fact in itself is taken into account, and a patient can only be considered stable if the EDSS score (the substitute measure to progression) is stable beside the absence of relapses and MRI progression [30]. However, these above-mentioned studies show that progression is largely independent from the EDSS score, so its measurement is hardly enough as evidence; thus, relying on it too heavily may give the clinician a false sense of security regarding the effectiveness of treatment.

The question then arises as to what are those clinical signs that could pose as “red flags” and draw the attention of a physician to this silent progression? Apart from the above-mentioned functional limb tests, there are severe consequences of MS, which are usually not routinely measured, prevalent and can pose serious deficits to patients with MS. These are the pathopyschological symptoms of the disease (cognitive impairment, fatigue, depression), of which we aim to give short overview in the next couple of paragraphs.

### 3.1. Cognitive Impairment

Cognitive impairment (CI) was long thought to be a rare aspect of MS and to only appear after long disease duration in the form of moria and euphoria. In the last three decades, this hypothesis was overturned: it was found that CI is among the most common symptoms of MS with prevalence values up to 65–70% [31,32]. It can appear as early as the CIS and radiologically isolated syndrome (RIS—practically accidental MRI findings suggestive of MS without any apparent clinical manifestation of the disease) states of the disease and can even hallmark the conversion to clinically definitive MS [33,34]. Yet, studies show it to be the most prevalent among patients with progressive disease courses: while the prevalence is usually around 50% in RRMS patients—as evidenced by our own, large-sample epidemiological study based on data from more than 500 patients—some assessments show it to be over 80% in patients with SP disease course [35,36].

CI in MS patients does not equal dementia and often, it is not as readily apparent as somatic symptoms [37,38]. Yet, it is a greatly important determinant of the patients’ quality of life: some assessments show it to be the most important reason for unemployment, social isolation, divorce and even the loss of driving capabilities [39]. Thus, it needs to be monitored regularly with tools preferably not requiring specialists and lengthy assessments, making it available in the clinical setting. The most commonly used assessments for this are the symbol digit modalities test (SDMT), the paced auditory serial adding test (PASAT) and the brief international cognitive assessment for multiple sclerosis battery (BICAMS) consisting of the SDMT, the brief visuospatial memory test (BVMT-R) and the California verbal learning test (CVLT-II) [40,41]. These tools are sensitive, can be administered rapidly and can reliably show the change in the patient’s cognitive state [40,41].

Despite the accumulating data that suggest it can appear rapidly during relapses or can even be the sole symptom during a relapse (hence the name “cognitive relapse”) followed by partial recovery, usually, it appears and progresses silently, independently from any relapses or somatic manifestations [42,43,44,45]. It seems that it is progressive in nature as a consequence of the disease as well: data show that once it is confirmed, there is very little chance for improvement [46]. These data and the fact that the highest prevalence of CI is among progressive patients suggest cognitive impairment to be a measurable sign of neurodegeneration rather than a simple consequence of inflammatory processes.

### 3.2. Other Pathophysiological Symptoms: Fatigue and Depression

Fatigue is the most common symptom of MS that virtually affects all patients during their lifetime [47,48]. Despite this frequency, it is even hard to define it, with several possible definitions in use. Yet, all these definitions address three underlying issues: (1) asthenia/daytime tiredness, (2) pathological exhaustibility and (3) worsening of symptoms due to stress [49]. Nowadays, fatigue is divided into two major categories: the perception of fatigue and objective performance fatigability [50]. As the latter is more measurable as an observable decrease in some kind of activity, the perception of fatigue is much harder to quantify as a result of being a subjective feeling. It is usually measured through self-reported questionnaires in the clinical setting, which makes it quantifiable and reproducible despite its subjective nature [51]. Depression is a major symptom of MS as well. Its prevalence differs greatly throughout the many epidemiological studies dedicated to it, yet all seem to agree that it affects a substantial portion of patients—with some data showing rates over 70% [52]. The differences are mainly due to different methodologies: studies utilizing hard, clinical diagnosis made by a psychiatrist usually report lower frequencies than assessments using self-reporting questionnaires [52]. Whatever the way of measurement, both psychological symptoms are, however, reported to be the worst consequence of MS by the patients [53]. Furthermore, in one of our works, we found on a large sample that fatigue and depression are the two most important determinants of the patients’ quality of life (QoL); no other symptom can affect QoL on such a diverse level [54].

### 3.3. The Problem of “Benign” MS

The term “benign” MS was defined as patients with disease duration ≥15 years and EDSS score ≤3 points, thus being in a relatively good physical condition despite a relatively long disease duration. The concept was abandoned with the arrival of the new phenotypic classification; however, the term is still in use sometimes, thus needing clarification. Assessments show that those patients with “benign” MS followed up for another 10 years are displacing the signs of the progressive disease and are in a much worse condition. This is because it seems there are great differences between individuals reaching the milestone of EDSS 3 points, which we consider to be the boundary of the appearance of irreversible neurological damage. Yet, after this phase, every patient “walks the same path” so to say, as the time between EDSS 3 points to EDSS 6 points is virtually the same in every patient. In the first phase, it may seem that the disease is “benign” as relapses are less frequent and the physical state is acceptable, yet the PIRA is not dormant. Nearly ¾ of “benign” MS patients show signs of fatigue and almost half of them have depressive symptoms [55]. Studies differ, but up to 47% of the “benign” patients have some degree of CI with detailed evaluations [56]. These data—combined with the earlier reviewed data on PIRA and PIRMA—clearly show that there is no such thing as “benign” MS, just symptoms of silent progression and neurodegeneration that are not routinely assessed.

However, it is important to understand that even these symptoms—as was shown above—are sometimes hard to be quantified or measured. Furthermore, sometimes they are only apparent after a time and can be subclinical or present, but not yet measured. Thus, there is a great need for reliable biomarkers—both imaging and on the cellular/molecular level, that are able to quantify these silent degenerative processes and to warn for timely intervention—even in the absence of clinically apparent changes.

## 4. Biomarkers

Biomarkers were defined as “any substance, structure, or process that can be measured in the body or its products and influence or predict the incidence of outcome or disease” by the World Health Organization (WHO) in 2001 [57]. Per this definition, any measurement can become a biomarker if validated, but a good biomarker needs to fit certain criteria: it has to be (easily) reproducible and cheap; it has to have good specificity and sensitivity; it needs to produce fast and reliable results and needs to be attained through minimal (or non-) invasive means [57]. As the CNS is probably the most separated part of the human body (in sense of physical, chemical and biological reachability), it is perhaps the hardest to develop biomarkers for the measurement of its processes. The lack of biomarkers is a pronounced problem in MS; despite the fact that a couple are widely used (e.g., MRI, oligoclonal bands in the cerebrospinal fluid (CSF), John Cunningham (JC) virus, etc.) for the diagnosis and some safety issues, biomarkers for the evaluation of disease severity or possible future prognosis—particularly in regard to the neurodegenerative processes—are seriously lacking. As we arrived in the new era in the management of MS with newer and more effective disease modifying therapies (DMTs) readily available and the treatment choice highly depending on the early severity of the disease (both activity and progression), the need for the earliest possible prediction of this severity and the exact follow-up of the drug’s efficacy is highly desired and important. These hypothetical biomarkers could have a great impact on the treatment: not only they can guide the choice for the best therapy at the beginning but can facilitate therapeutic interventions at the earliest sign of ineffectiveness that cannot be measured in any other way. Yet, sadly, to date, there is no validated, easily accessible, and widely utilized biomarker of the aforementioned kind available. However, a change seems to be arriving in the last couple of years: more and more candidate biomarkers are studied for this very purpose, both in the molecular (serum, CSF) “realm” and through imaging (MRI, optical coherence tomography (OCT)) means. In the next couple of paragraphs, we aim to give a short, comprehensive review of the ones that are used, or possibly will be used in the future in multiple sclerosis.

### 4.1. Imaging Biomarkers

#### 4.1.1. MRI

MRI has become the cornerstone of the diagnosis and follow-up of MS patients in the last few decades. It is the single most important diagnostic tool and the diagnosis can be made after only one clinical event and one MRI measurement [2]. There are several studies both in the past and recently that report the efficacy of MRI in the measurement of the activity of the disease, even as a prognostic factor for future severity of the disease [58,59]. However, these routinely assessed markers (the T2-hyperintense lesion load, T1-hypointensity, gadolinium enhancement) are measures of inflammation and do not correlate well with neurodegeneration. As a recent study reported, the progression of the disease is largely independent from these MRI parameters [60]. However, there is a specific MRI marker which correlates well with neurodegeneration: atrophy. Both global atrophy and atrophy of specific regions show significant connection to the neurodegenerative process’ clinical signs of MS.

The global brain atrophy rate in MS patients is much higher than in the normal population (0.05–0.3%), reaching 0.35–1.35% annually [61,62]. The global atrophy rate correlates well with the EDSS score and disease progression up to 10 years [63].

Recently, the measurement of gray matter atrophy became a diagnostic cornerstone, since it correlates with psychopathological symptoms in MS; therefore, it should be a therapeutic goal of new treatments [64,65]. The mechanism of gray matter atrophy is not fully understood. According to Jehna et al., periventricular lesions correlate with cortical atrophy, which is based on a cerebrospinal fluid-mediated pathological process [66]. The other mechanisms of cortical atrophy include remote axonal transections, reduced cortical input and the reduction in synaptic density [67]. In MS patients, magnetic resonance spectroscopy showed low *N*-Acetylaspartate levels in the normal-appearing white matter as a sign of axonal loss or dysfunction, which also correlates with brain volume loss [68]. Based on these data, Tóth et al. examined whether the periventricular white matter lesions have a connection with gray matter atrophy [69]. They used diffusion tensor imaging to detect white matter deterioration and found remarkable alterations in diffusion parameters, which suggests demyelination both in lesion burden periventricular white matter and in the normal-appearing white matter. These diffusion alterations are the best predictors of the detected significant brain atrophy. All in all, gray matter atrophy has been shown to be the best predictor of cognitive dysfunction, fatigue and depression as well [70,71,72].

The most sensitive MRI marker of neurodegeneration is thalamic atrophy among specific regional atrophies, which occurs already in the early stage of MS and declines over the disease period. The thalamus has numerous cortical and subcortical connections; the axonal transection caused by white matter lesions can be detected in the thalamus and its pathways. Other mechanisms, such as iron deposition, demyelinating lesions in thalamus, or reduced neuronal density in non-lesional thalamic tissue, also contribute to thalamus atrophy. Therefore, the detection of thalamic atrophy helps to estimate MS-related damage in the entire CNS [73,74]. Azevedo et al. performed a study on thalamic atrophy and its clinical relevance in MS and found significant correlation between atrophy and EDSS and all of the multiple sclerosis functional composite (MSFC) components [75].

When these results are taken into consideration, it is not surprising that the concept of NEDA evolved from NEDA-3 to NEDA-4 and measuring whole brain atrophy (WBA) became the new “member of the club”. However, there are certain difficulties with the standardized measurement of WBA (which stretch the frame of our review, thus we cannot give an in depth analysis of it), so the regular follow-up of WBA is still not routinely undertaken. Yet, it should provide a hard imaging biomarker for clinicians in the upcoming years.

#### 4.1.2. Optical Coherence Tomography

A novel and simple method to monitor neurodegeneration is OCT, which measures the thickness of the retinal nerve fiber layer (RNFL) and the macular ganglion cell layer (mGCL). In RNFL, the demyelinated CNS axons are accessible. OCT is a non-invasive, quick and easy to use method with high resolution, which involves the usage of near-infrared light to examine the retina. In MS patients, the thickness of GCL and RNFL detected by OCT was found to be lower than in the healthy control. Several studies confirmed that OCT alterations are markers of neurodegeneration, and are also related to physical disability, cognitive deficits and cerebral atrophy [76,77].

### 4.2. Molecular Biomarkers

Molecular biomarkers in MS can be classified several ways, but there are two criteria which are always highlighted: (1) are they markers of activity (inflammation) or progression (neurodegeneration) or (2) can they be measured only in the CSF or in the serum as well. The latter distinction is highly important, as a good biomarker needs to be collectable through minimal invasive means as a series of lumbar punctures (LPs) for any means is hardly a desired process, neither for the patient nor for the doctor.

#### 4.2.1. Neurofilaments

Neurofilaments (NfLs) are neuron-specific cytoskeletal components most abundantly found in axons, with their most important function being the structural support and thus maintaining the shape and size of the axons [78]. NfLs are a subtype of intermedier filaments and are comprised of three subunits: heavy, medium and light chains—based on their molecular weight [79]. In the last few years, the light chain became the most promising candidate as a biomarker. As NfLs can solely be found in the cytoplasm of neurons, it is a specific marker of axonal/neuronal damage both in the CNS and in the peripheral nervous system (PNS). Yet, as NfLs are released to the CSF and the blood regardless of the type of injury the neuron/axon acquired, all diseases which may damage neurons elevate the level of NfL in body fluids.

At the beginning, studies mainly focused on the CSF, as the first and second generation tools (immunblot and regular enzyme-linked immunosorbent assay (ELISA)) were only able to reliably detect NfLs there, while amounts in the serum are orders of magnitudes lower. Nowadays, first with electrochemiluminscent assays (ECL), then with single molecular arrays (SiMoA), serum (and plasma) levels can be readily measured [80]. As the levels of NfLs in the serum correlate well with the levels in the CSF, continuous monitoring can be achieved through simple blood sampling without the need for the invasive method of LP [81]. This led to the “shift” of interest toward serum NfL levels as a potential biomarker.

Regarding MS, NfLs have recently become the focus of interest and the majority of the studies were conducted on CSF at the beginning; then, more and more data arose regarding serum NfL levels. Assessments of patients at risk for developing MS (after optic neuritis (ON), CIS and RIS patients) showed somewhat controversial results, yet most evaluations agreed that elevated levels of NfLs in the CSF is an independent—if not a strong—risk factor for converting into clinically definitive MS [82,83,84,85]. Several studies observed that CSF and serum NfL levels are generally higher in RRMS patients than in healthy controls, and this difference can be tenfold if the evaluation is carried out during a relapse [78,86,87]. Regarding disease progression, the picture is not as clear so to say, but there are studies implying that more elevated NfL levels at the beginning can predict a EDSS progression at 5 and 10 years, and an earlier conversion into SPMS [88,89]. An assessment carried out by our colleagues in the department found CSF NfL levels to be the best predictor of future disability [90]. Several studies, however, showed the clear association of NfL levels in the CSF and serum with MRI lesion burden, the development of new lesions and Gd-enhancement, or the change in functional connectivity [85,91,92,93,94,95,96]. Serum NfL levels were successfully utilized to monitor the treatment response for almost all approved DMTs—regardless of the disease course [80,91,97,98,99,100].

Despite these really encouraging data, there are still obstacles in the way of the general utilization of NfL levels in everyday medicine. There are several physiological processes (e.g., age) that can greatly influence NfL levels as well as other pathological processes, through presently unknown means, outside of the nervous system—e.g., hypertension—which can make the interpretation challenging [101]. A recent publication even established that both acute and continuous exercise can decrease the levels of NfLs in the body fluids, further worsening the accuracy of its measurement [102]. The measurement of intraindividual change (probably due to the extremely low levels) of serum NfLs is still often inaccurate, rendering it unusable for individual follow-up, while monitoring through the CSF cannot be encouraged due to the need of serial LPs. A reference laboratory still could not be established, nor validated normative data given, and there are still different methodologies and different kits in use, so the results in different laboratories cannot be reliably compared. All in all, despite these challenges, NfLs seem to be a good and highly utilizable biomarker candidate, which has the potential to become part of everyday practical neurology in the near future.

#### 4.2.2. Other Possible Molecular Biomarkers

Apart from NfLs, there are several other candidate molecular biomarkers continuously researched in case of MS. A couple of them have shown particularly encouraging results and possibly will be utilized in the future.

Osteopontin (OPN) is an extracellular matrix protein which is involved in the pathology of several inflammatory diseases [103]. However, it has a diverse role, thus is expressed during both physiological and pathophysiological (from bone remodeling to vascular diseases) processes as well. It is abundantly expressed in immune cells (T-cells, dendritic cells, NK-cells) and works by increasing the production of proinflammatory and inhibiting anti-inflammatory cytokines [103]. It was found that the overall level of OPN was higher in MS patients than in healthy controls both in the CSF and the serum [103]. It seems that the concentration is the lowest in CIS, and the highest in RRMS patients, with the levels in progressive patients in between [103]. Among RRMS patients, active patients have a significantly higher concentration in both CSF and blood than in patients with stable disease [103]. A study conducted by Szalárdy et al. in our department found that OPN in CSF might be an independent marker of disease severity [104]. Some data imply that the effect of natalizumab can be explained partly by the fact that it inhibits the connection between OPN and α4-integrin, and thus decreases inflammatory processes [105]. Hence, a measurable decrease in OPN levels can be observed and it was shown to be connected with better cognitive performance of the patients [106]. It seems that overall, OPN can be a useful biomarker in the future for monitoring inflammation, neurodegeneration and possibly some therapeutic interventions as well.

Peroxisome proliferator-activated receptor-*γ* (PPAR-*γ*) is a subtype of the nuclear hormone receptor superfamily of ligand-activated transcriptional factors. It is mainly involved in glucose and lipid metabolism, but several studies showed it has an important role in the regulation of immune responses [107]. PPAR-*γ* was shown by several studies to inhibit the expansion of auto-aggressive T-cells towards the CNS [108,109,110,111,112]. It was also established that its activation inhibits the differentiation of CD4+ T-cells towards the Th17 phenotypes, and decreases the expression of IL-17 in the CD4+ T-cells infiltrating the CNS in experimental autoimmune encephalomyelitis (EAE) mice models, thus presenting strong anti-inflammatory qualities [111,112,113]. Several other animal studies using genetically deficient animals or the pharmacological suppression of PPAR-*γ* resulted in the aggravation of the severity of the EAE in the animals, further suggesting an important modulatory role toward anti-inflammatory responses of the molecule [111,113,114]. Quite a few evaluations have shown that the molecule has strong neuroprotective properties (both on neurons and oligodendrocytes) and through promoting the differentiation of oligodendrocytes, it has an important role of promoting remyelination [115,116,117,118,119]. Not so long ago, Szalárdy et al. demonstrated a clearly elevated level of PPAR-*γ* in the CSF of MS patients as compared to healthy controls, suggesting a compensatory mechanism against the inflammatory processes and an association with more severe disease activity [120]. All in all, the molecule is on one hand, a promising biomarker, and on the other, a possible therapeutic target—but this possibility extends the boundaries of our review and was elaborated on elsewhere in detail [121].

## 5. Kynurenines as Biomarkers for Progression and as Possible Therapeutic Targets

### 5.1. Kynurenines and the Kynurenine Pathway: Neuroactive Metabolites

**Figure 2:**
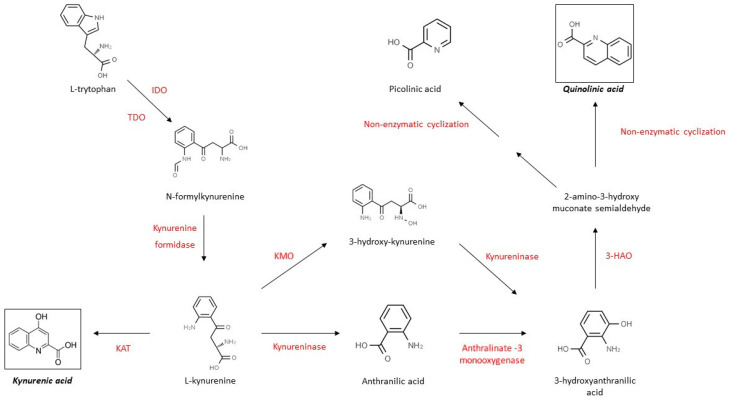
Metabolism of tryptophan: the kynurenine pathway. IDO, indolamine 2,3-dioxygenase; TDO, tryptophan 2,3-dioxygenase; KAT, kynurenine aminotransferase; KMO, kynurenine 3-monooxygenase; 3-HAO, 3-hydroxyanthranilate oxidase.

Kynurenines are a collective name given to several molecules that are the product of the metabolism of tryptophan (Trp). Trp is an essential amino acid scarcely found in humans, and a precursor for several essential proteins such as nicotinic acid, nicotinamide adenine dinucleotide (NAD+), serotonin or melatonin [122]. Roughly 95% of its metabolism is through the primary, kynurenine pathway (KP), see Figure 2, in both the PNS and the CNS, and it is mainly bound to glial cells after the uptake of Trp through the BBB by the competitive l-type amino transporter [123,124]. The rate-limiting enzyme of the pathway is indolamine 2,3-dioxygenase (IDO) in the nervous system and tryptophan 2,3-dioxygenase (TDO) in the liver that converts Trp to *N*-formyl-l-kynurenine [125,126]. This step is followed by the catabolism of *N*-formyl-l-kynurenine to l-kynurenine (l-KYN), the central molecule of the KP by the enzyme formamidase [126]. Depending on the tissue, three separate enzymes can metabolize l-KYN (kynurenine aminotransferase (KAT), the kynurenine 3-monooxygenase (KMO) or the kynureninase) into kynurenic acid (KYNA), 3-hydroxy-l-kynurenine (3-HK) or anthranilic acid (AA), respectively [126]. The latter two molecules can be further metabolized into 3-hydroxyanthranilic acid (3-HAA) and then by the 3-hydroxyanthranilate oxidase (3-HAO) to quinolinic acid (QUIN), a primary neurotoxic precursor of NAD+ and nicotinamide adenine dinucleotide phosphate (NADP+) production [126].

#### 5.1.1. Kynurenic Acid

KYNA is one of the “end products” of the KP and—in contrast to the other metabolites—it is mainly synthetized in astrocytes rather than microglial cells [127,128,129,130,131]. KYNA in the CNS is almost exclusively synthetized there as it cannot cross the BBB [126,132]. KNYA is a competitive antagonist of the ionotropic glutamate receptors (*N*-methyl-d-aspartate (NMDA), α-amino-3-hydroxy-5-methyl-4-isoxazolepropionic acid (AMPA), and the kainite). It has the highest affinity to the NMDAR through the strychnine-insensitive glycine-binding site at low and at the glutamate-binding site at high concentrations [133,134]. KYNA also has a dual effect on AMPA receptors which is concentration-dependent (it acts as an antagonist only in higher concentrations) and is also an endogenous agonist to the orphan G-protein coupled receptor GPR35 [135,136,137]. By binding to these receptors, KYNA is able to inhibit excessive Ca^2+^ influx into the neurons—thus protecting them from a neuronal death [126]. In addition, more and more data arose that KYNA is a potent antioxidant of the CNS at physiological conditions [138]. Both in vivo and in vitro evidence accumulated that KYNA can firstly halt lipid peroxidation, and secondly act as a scavenger for reactive oxygen species (ROS) which further increases its neuroprotective capabilities [138,139].

#### 5.1.2. Quinolinic Acid

As opposed to KYNA, QUIN has strong neuroexcitatory properties which can be attributed to several mechanisms (Table 1). It inhibits glutamine synthase and glutamate uptake at pathophysiological concentrations, may cause lipid peroxidation and—in the presence of Fe^2+^—promotes the formation of ROS [134,140,141]. The most important mechanism is, however, its extremely specific competitive agonism of the NMDAR containing the subunits NR2A, NR2B, and NR2C [142]. Through this specific agonism, it exerts the opposite effect to KYNA: it creates glutamatergic excitotoxicity through promoting excess Ca^2+^ influx resulting in neuronal death [126]. The NMDA receptors’ sensitivity to QUIN is inequivalent and is based on their subunit composition; thus, QUIN exerts different levels of toxicity in different sites of the brain [143,144,145,146,147].

### 5.2. The Role of the Kynurenine Pathway in Neurodegenerative Conditions

The KP has been implicated in numerous physiological processes: the IDO1 enzyme is a potent immunosuppressor; Trp-metabolites have multiple roles regarding the physiological state of the gut microbiome; the KP is indicated in normal embryonic development and are important regulators of the physiological ageing process [148,149]. Furthermore, the KP has become a key area of research regarding neuropsychiatric and neurodegenerative conditions due to accumulating evidence of the disturbances of KP metabolites and enzyme processes in these diseases.

#### 5.2.1. Kynurenines in “Classical” Neurodegenerative Diseases

Classical neurodegenerative diseases all characteristically display progressive neuronal loss as the key pathophysiological process, yet there is another connection between them: the KP and kynurenine metabolites seems to play an important role in these conditions.

##### Huntington’s Disease

Huntington’s disease (HD) is an autosomal dominantly inherited neurodegenerative condition characterized by CAG-triplet expansions within the huntingtin gene (HTT) resulting in a polyglutamine stretch in the HTT protein. There are plenty of studies assessing the role of the KP in HD. In the early stages of the disease, both QUIN and 3-HK levels are elevated in the striatum and cortex of the patients, while at the same time, KYNA levels are markedly decreased both in the brain and the CSF [150,151,152]. An increased ratio of l-KYN/Trp in the blood of HD patients is present, which might be explained by the upregulation of the IDO1 transcription observed in animal models [153,154,155]. These findings suggest that an abundance of l-KYN present in HD leads to high activity of the QUIN and low activity of the KYNA arm of KP, implying a possible causative role in the disease [139].

##### Alzheimer’s Disease

Alzheimer’s disease (AD) is the most common neurodegenerative disease characterized by progressive dementia. It is of multifactorial etiology and the characteristic pathology of the disease is the accumulation of misfolded *β*-amyloid plaques (A*β*) in the brain and the intracellular phosphorylated tau-protein neurofibrillary tangles. There are plenty of data proving the role of the alteration of the KP in the pathophysiology of the disease [156]. Compared to healthy controls, there is an elevated ratio of l-KYN/Trp in the blood and CSF of AD patients parallel to increased levels of IDO activation in the brain tissue [157,158]. Several evaluations observed that KYNA levels are lower, while QUIN and 3-HK levels are significantly higher in the serum and CSF of AD patients as compared to healthy controls [139]. In a study, exposing human neurons to QUIN resulted in the upregulation of genes connected to tau phosphorylation, implying a path in which neurofibrillary tangles can be formed [159]. These findings clearly demonstrate that the alterations of the kynurenine metabolism contribute to AD pathology.

##### Parkinson’s Disease

Parkinson’s disease (PD) is the second most common neurodegenerative disease of the CNS, characterized by the loss of dopaminergic neuron deficit and loss. Similarly to other neurodegenerative conditions, the KP shows alterations in PD as well. It was shown in animal studies that increased levels of QUIN and 3-HK can be found in both the brain and plasma [160]. Both KAT-I and KAT-II activity was lower in the plasma of PD patients, resulting in a lower level of KYNA observed [161]. Some studies found reduced levels of KYNA in the cortical regions and the basal ganglia of PD patients [162]. Elevation in both the 3-HK/KYNA and the QUIN/KYNA ratio was observed in PD patients [163]. In animal models, the bilateral injection of KYNA lessened the motor symptoms of monkeys [164]. It seems that the inhibition of KAT-II can increase the level of dopamine in the striatum and the co-administration of KYNA can expand the longevity of this effect [165]. All in all, it seems that the KP is a viable candidate in PD both as a biomarker and as a therapeutic approach.

#### 5.2.2. Kynurenines in the Retina and Its Diseases

The KP—mainly the KAT enzymes and KYNA—plays an essential role in the development of the retina. It was shown in animal models that the level of KYNA and KAT activity is the highest in the perinatal period and rapidly decreases after birth [166,167]. As NMDAR is crucial in synapse development and the migration of neurons, KYNA seems to provide an endogenous antiexcitotoxic protection before and during birth and the quick decrease assures that it would not interfere with postnatal, essential receptor functions [168]. It was also implied by evaluations that through the different spatial distribution and temporal expression of the KAT enzymes and KYNA content, the KP is a key mediator in controlling apoptosis in the retina during early development [169,170]. Knowing this, it is not surprising that the KP was implicated in the process of retinal ganglion cell (RGC) loss. It seems to be affected by NMDAR—and AMPA and kainate receptors as well [171,172,173]. After NMDA injection into the retina, a marked increase in KYNA was observed—corresponding to a heightened neuroprotection at the beginning, and a few days later both KYNA levels and the number RGCs decreased significantly [174]. In the DBA/2J mice model for glaucoma, KYNA concentrations changed parallel to a time-dependent RGC loss [175]. Immunohistological assessments found the decreased cellular expression of KAT enzymes in the same mice model [175]. All in all, these evaluations found that the decrease in KAT enzyme activity and KYNA levels in the retina imply a possible role of the KP in degenerative diseases such as glaucoma and optic neuropathy.

#### 5.2.3. Mood Disorders and Suicide

Trp metabolism was found to be a major contributor to the development of several psychiatric conditions on the molecular level. However, major depressive disorder stands out, as it was causally linked with the KP [149]. It seems that the increased metabolism through the 3-HK pathway leads to increased levels of QUIN over KYNA in the brain, leading to neurotoxicity [176]. The same mechanism was found in suicidality [177,178]. It seems that the upregulation of IDO1 is a key point in this process, yet it is unknown why the 3-HK pathway is so unequivocally upregulated [149].

In summary, the KP plays a key role in the pathomechanism of several neurodegenerative diseases and conditions. Interestingly, we can find a lot of symptomatic connection between such diseases and MS. First, a pivotal symptom of all the aforementioned “classical” diseases is cognitive decline, which is the direct consequence of neurodegenerative processes. CI is one of the most substantial symptoms of MS as well, and it is clearly associated with neurodegeneration (as was elaborated above). Mood disorders are among the most common and debilitating symptoms of MS and optic neuropathy is considered a classical—often initial—symptom of the disease—with retrograde axonal loss being a classic long-term consequence of retrobulbar neuritis in MS. Furthermore, in all these classical neurodegenerative conditions, brain atrophy is a prominent feature, while it is becoming a possible biomarker in MS—just like the neurodegeneration measured in the retina by the OCT. With this knowledge in mind, these symptomatic and imaging connections point to a common mechanism, and this connecting point might be the KP, as all the described processes can be directly linked—in some cases causally—to the kynurenine pathway and its disturbances.

### 5.3. The Role of the Kynurenine Pathway in the Neurodegenerative Processes of Multiple Sclerosis

The role of the KP has been well-established in the pathogenesis of MS both in animal models and in human subjects as well. We recently elaborated on the role of KP in the immunodysregulation and pathogenesis of MS, in a review by Biernacki et al. [126]. Now, we summarize the evidence which points to the central role of the KP in the neurodegenerative process in MS.

#### 5.3.1. In Vitro Results and Animal Model Studies

As was mentioned above, oligodendrocyte apoptosis can be the initial step in the pathogenesis of MS. QUIN is a potent neurotoxin and is produced both in microglia and in macrophages—albeit in quantities an order of magnitude lower in the former [179,180]. However, a hallmark of MS lesions is the presence of both cells during the active phases—in part due to the damage to the BBB, and thus the invasion of macrophages into the CNS [181]. QUIN was proven to induce oligodendrocyte apoptosis in pathophysiological concentrations in vitro [182]. QUIN can also induce axon-sparing lesions when introduced to rat brain slices in pathophysiological conditions [183]. In rats with EAE, QUIN-induced oligodendrocyte cell death in the spinal cord was observed by multiple studies [182,184]. QUIN is a potent agonist of NMDAR but also acts on AMPA and kainate receptors and induces excitotoxicity through all receptors. This was evidenced by a study with an EAE mouse model, where the blockade of AMPA and kainate receptors had no effect on the lesion size and inflammatory processes [185]. A later study was able, however, to rescue oligodendrocytes completely from injury by inhibiting all three receptors [186]. It was shown in 2014 though, that oligodendrocytes might be able to catabolize QUIN and therefore have a role in the defense against excitotoxicity, but the excess of their capabilities in this manner are still unknown—presumably not decisive [187]. QUIN, however, acts not only on oligodendrocytes. It was shown that QUIN produced by macroglia and macrophages induces apoptosis in astrocytes as well [188]. It is of utmost importance, as astrocytes are the primary “guardian” cells against excitotoxicity by producing KYNA and by being able to catabolize QUIN by quinolinate phospho-ribosyltransferase [127,189]. However, astrocytes are only able to catabolize a low amount of QUIN because the saturable activity of the enzyme is low [127,189]. Thus, by the combined effect of the two processes, QUIN can overcome the effects of the quinolinate phospho-ribosyltransferase and “destroy the defenders” so to speak, thus inducing an excitotoxic feedback loop. QUIN, however, can have direct effects on the neurons as well. Chronic exposure of the rat striatum to QUIN induced cognitive dysfunction in the animals [190]. Human neurons in vitro treated with pathophysiological doses of QUIN resulted in apoptosis [191,192]. Furthermore, it was found that QUIN inhibits the neutralization of ROS and other free radicals in cells, changes the glutathione redox potential, and depletes superoxide dismutase activity; all in all, it disrupts the mitochondrial function severely, thus further inducing neurodegeneration and cell death [193,194,195,196,197].

Numerous studies were conducted using the EAE model assessing the role of the enzymes in the KP. Increased IDO-1 activation with an elevated Kynurenine/Trp ratio was observed in the brain and spinal cord of mice with EAE that was accompanied by decreased levels of interferon-*γ* (IFN-*γ*) levels, thus implying a decrease in inflammatory activity [198]. The inhibition of the enzyme (either by genetic deficiency or pharmacological blockade) on the other hand resulted in a more severe clinical course of the disease [198,199]. However, in a recent evaluation, the timely inhibition of IDO-1 enzyme—after the initiation of immune tolerance—significantly decreased the severity of EAE compared with the untreated animals [200]. It shows that IDO-1’s role may change in the pathogenesis of MS during time and chronic, low level activity tips the balance toward neurotoxicity rather than protection [200].

The deficiency of the other rate-limiting enzyme, TDO, has resulted in neuroprotection in the spinal cord of EAE mice; however, this histological protection was not translated into clinically measurable levels [201].

Another important enzyme in the KP, KMO, was implicated in the pathogenesis as well. Some studies found that the upregulation of the enzyme leads to a higher level of 3-HK and QUIN production to neurotoxic levels in the spinal cord of EAE rats [126]. Yet, the inhibition of KMO with Ro 61-8048, a selective inhibitor, lowered the level of neurotoxic molecules and elevated KYNA levels [199]. Yet, this study did not find differences in the severity of the EAE between the treated and untreated animals [199]. However, in the same study that measured the effect of timely IDO-1 inhibition, the authors found an even more robust decrease in severity in the Ro 61-8048-treated animals than with IDO-1 inhibition, further underlining the role of KMO in neurotoxicity [200]. High KMO activity was found in subpial, subependymal and perivascular macrophages in EAE rats, implying that the source of the neurotoxic kynurenine metabolites is partly these peripheral cells allowed by the deficiency of the BBB [198,199]. As both microglia and macrophages continuously release pro-inflammatory cytokines, which in turn result in the production of more and more QUIN and other toxic KP metabolites, the whole process becomes a positive feedback loop, further promoting neuronal injury and death. On the other hand, in the inhibition of KMO in animal models of neurodegenerative diseases, a clear neuroprotective effect could be observed, with better cognitive and mood outcomes [202].

Other than EAE, the most often utilized animal model for MS is the cuprizone model. A recent study found, however, that the use of cuprizone significantly tips the balance of the KP to neurotoxicity by significantly lowering KYNA levels both in the brain tissue and the plasma [203]. The significance of this finding is not completely clear yet, but further investigations are needed to determine the reason and the consequences.

#### 5.3.2. The Kynurenine Pathway Metabolites in Multiple Sclerosis

The two basic clinical hallmarks of MS are based on the course of the disease: the relapsing–remitting and the progressive disease courses. The clinical phenotypes, however, reveal differences in the underlying pathomechanism as well: while in the relapsing phase, the episodic, flaring-up inflammation is the dominant pathology, during the progressive phases, the steady neurodegenerative processes dominate the disease—while of course, both processes contribute to the overall pathology. These two differing processes theoretically should be represented in the cellular and molecular make-up of the patients in vivo. As the KP is a key regulating pathway in both neuroinflammation and neurodegeneration, the differences in the metabolite and enzymes could prove to be such targets.

The first major findings regarding the KP in MS patients was that Trp levels were significantly lower in both the CSF and the serum of the patients—implying the measurable activation of the KP during the disease [204]. Several studies since corroborated these results [205,206]. There were evaluations proving that KYNA levels are elevated during acute relapses, while they are lower in the remission phase [207,208]. Other studies observed decreased KYNA and picolinic acid levels, while finding an increase in QUIN levels in MS patients CSF [209]. A study even found a causal connection between increased QUIN levels and pathologic tau-phosphorilation [210]. Hartai et al. found elevated levels of KAT-I and KAT-II enzymes in the red blood cells of MS patients, while the two enzyme levels were significantly decreased in the brain tissue of the patients in another study [211,212].

A pivotal recent study evaluated the metabolomics profile of MS patients, and yielded highly exciting results. This study was able to build a highly sensitive (up to 91%) model for the prediction of MS courses with six predictors, and the three most relevant were KYNA, QUIN and Trp [213]. It also showed that the lowest rates of the neuroprotective metabolite KYNA can be found in the progressive disease courses [213]. Another high quality study from 2019 found that the levels of Trp metabolites KYNA, 5-hydroxytryptophan, 5-hydroxyindoleacetate, and *N*-acetylserotonin differ greatly in the CSF of SPMS patients as compared to RRMS patients [214]. Recently, Rajda et al. evaluated markers of both neuroinflammation and neurodegeneration in the CSF of RRMS patients. They found with receiver operating characteristic (ROC) curve analysis, that the best predictors for disease severity were NfLs, QUIN and neopterin, which all were elevated in the serum of the patients as well, compared to the members of the control group [90]. A thorough evaluation assessing the possible differences between disease courses found that the levels of Trp metabolites in the overall MS group did not differ from controls; however, assessing the different courses separately yielded very different results. The levels of Trp and KYNA are decreased in the SPMS group, while the levels of QUIN and the QUIN/kynurenine ratio is elevated in RRMS patients, while every metabolite level is increased in the PPSM group—markedly differing from RRMS and SPMS patients and showing surprising similarity to other inflammatory diseases, such as systemic lupus erythematosus (SLE) [215]. Another interesting evaluation measured the levels of Trp metabolites in depressed MS patients. They were able to demonstrate higher KYNA/Trp and kynurenine/Trp ratios in patients, supporting the KP’s role in MS and depression [216].

All in all, accumulating evidence supports that the KP plays an important role in both the inflammatory and degenerative processes in MS (Table 2 and Table 3). The former is no surprise, as KP’s involvement in the initiation and the regulation of immune responses are well described. However, the measurable and significant changes in of the KP metabolites in processes directly linked with neurodegeneration prove that the KP’s role is even more widespread in MS than we earlier believed. This leads to the exciting conclusion that the external manipulation of the kynurenine pathway may prove to be a viable therapeutic strategy in all clinical courses of the disease in the future.

### 5.4. Therapeutic Capabilities of Kynurenines

#### 5.4.1. Preclinical Studies

The substantial neuroprotective effect of KYNA is obvious, evidenced by the mass of data reviewed in our articles in earlier paragraphs; thus, it would be a natural choice as a neuroprotective drug. The problem hindering its successful use is that KYNA administered to the periphery is almost completely unable to penetrate the BBB. This means that KYNA either has to be administered directly to the nervous system (which is, understandably, not a viable choice) or other molecules that act the same way but can cross the BBB readily must be found. Three methods have been proven to work in this manner: 1, several synthetic KYNA analogues have been synthesized and used in trials; 2, l-KYN can be administered, as it was proved to penetrate the BBB and its administration significantly raises KYNA levels in the CNS; 3, structurally similar molecules that have (some) similar effects on the KP can be found.

Synthetic analogues of KYNA have been produced and tested since the early 1990s [217]. Since then, new efforts are continuously made to increase the effectivity and safety of these molecules. There are several successful attempts reported in the recent past as well—several of those reports were by our research group [218]. It seems the most effective such derivatives are KYNA-amides, which are known to be capable of the selective inhibition of the NR2B subunit-containing NMDA receptors [219].

In preclinical (both in vitro and in vivo animal studies), these molecules have proven to effectively halt neurodegeneration and inflammation. In a recent evaluation, it was found that newly synthetized KYNA analogues can effectively decrease tumor necrosis factor-α (TNF-α) production in *Staphillococcus aureus*-induced cell cultures [220]. In another recent evaluation on *bdelloid rotifer* species, KYNA and its analogs increased longevity, reproduction, and growth in animals, while reducing the capacity and energy-dependent muscular activity at the same time [221]. Another molecule, 1-methyl-Trp, was found to effectively drive Trp-catabolism toward he KYNA path in both mice and ex vivo human blood cultures [222]. One particular KYNA analogue, SZR-104, was not only proven to effectively cross the BBB and halt epileptic seizures in mice but also was able to inhibit microglia activation in the brain of the animals [223,224,225]. The analogue reached a higher concentration level in these studies than KYNA did [223]. These studies are recent findings that—together with abundant earlier evaluations—show the promising efficacy of KYNA and its derivatives in the therapy of neurological conditions.

#### 5.4.2. Clinical Studies

There were, however, not only preclinical, but several clinical studies performed with other derivatives of KYNA, or with molecules with surprising structural similarity to it to examine the safety and the efficacy of such molecules in neurological (both degenerative and inflammatory) conditions. The detailed description of these studies exceed the scope of our review, but two recent review articles of our research group presented these studies in detail, with one of them particularly focusing on MS [126,134].

Here, we would like to review, however, another clinical study that was reported recently as a collaboration of Danish, Swede and Hungarian contributors [226]. It is a Phase I study that assessed the safety, tolerability and the physiological reaction to intravenous l-KYN administration in both rats and humans—the first evaluation to assess l-KYN administration in human subjects [226]. According to the study design, seven male Sprague Dawley rats were anesthetized then cannulated. First, calcitonin gene-related peptide (CGRP) was administered to the animals followed by l-KYN administration in increasing doses. At the end of the assessment, CGRP was administered again into their carotid artery. The change in the diameter of dural, pial arteries and blood pressure were monitored as endpoints. Twelve healthy people were also recruited for the study in Denmark. All participants were free from any somatic or psychiatric disease other than episodic tension headache. They were separated into two groups: the first consisted of six participants, all of whom received 50, 100, and 150 μg/kg l-KYN in a continuous intravenous infusion over 20 min on three days separated by at least 1 week. The second group also had six participants; however, they received 0.3, 0.5, 1, and 5 mg/kg l-KYN, also by continuously administered intravenous infusions, over 20 min on four days separated by at least 1 week. The endpoints were the blood flow velocity of the middle cerebral artery (VMCA), the left superficial temporal artery (STA) diameter, the left radial artery (RA) diameter, and the end-tidal partial pressure of CO_2_ (PetCO_2_). These were recorded before, 10 min after, and then every 20 min until 120 min following the beginning of infusions. All participants were measured for headache, and were required to complete a headache diary until after 24 h since the beginning of the infusion. l-KYN and kynurenine metabolites were then measured from both blood and urine samples up to 120 min after the infusion.

In the animals, the administration of l-KYN did not result in any significant change in any of the measured parameters. In the human participants, a mild headache developed in four out of six patients at the smallest dose, yet with the increase in the l-KYN dose, the headaches disappeared. The levels of l-KYN significantly increased until the peak point at 20 min after the infusion, then the levels started to drop exponentially. The half-life was 94 min. Only a trend-like change with no statistical significance was observed in the levels of KYNA and Trp in the blood and urine samples. The examiners found no difference in the four observed parameters (VMCA; STA and RA diameter and PetCO_2_).

It can be concluded that the administration of l-KYN is safe and tolerable up to 5 mg/kg doses without any adverse events whatsoever. It seems that the metabolism of l-KYN is slow, and even higher doses with longer follow-up periods may be needed in further investigations. Thirdly, it might be advantageous to measure KYNA levels in the CSF as well. All in all, this study further underlines the possibility of the development of a KP-derived drug in the near future. It shows that whichever way we endeavor to utilize KP metabolites (as analogues, in pro-drug form, etc.), it seems to be a valid approach. Further studies in the near future will surely report exciting outcomes regarding the KP.

## 6. Conclusions

More and more evidence emerges about the pivotal role of neurodegeneration in the pathomechanism of MS regardless of the phenotypical course of the disease. Biomarkers are on the verge of widespread use—either imaging or molecular in nature. The kynurenine pathway seems to be important both in the pathomechanism of neurodegeneration in MS and as a possible biomarker in the near future. Even more importantly, several pre-clinical and clinical evaluations paint kynurenine metabolites and analogues as potential neuroprotective therapies in the future of multiple sclerosis, heralding a new era of therapies capable of more than immune-modulation, but possibly halting (or even reversing?) sustained disability.

## Figures and Tables

**Table 1:** The antagonistic effect of kynurenic acid and quinolinic acid.

	KYNA	QUIN
Formation	mainly in astrocytes	mainly in microglia
Effect on NMDA receptors	antagonist	agonist
Effect on AMPA receptors	antagonist	-
Effect on Kainate receptors	antagonist	-
Effect on GPR35	activates	-
Effect on glutamate reuptake	-	inhibits
Intracellular kation (Ca^2+^) influx	inhibits	promotes
Lipid peroxidation	inhibits	promotes
Effect on ROS formation	scavenges ROS	promotes formation

KYNA, kynurenic acid; QUIN, quinolinic acid; NMDA, *N*-methyl-d-aspartate; AMPA, α-amino-3-hydroxy-5-methyl-4-isoxazolepropionic acid; GPR35, orphan G-protein coupled receptor; ROS, reactive oxygen species.

**Table 2:** Kynurenine pathway metabolites and enzymes—results of in vitro and animal studies.

QUIN	KMO	IDO-1
Induces oligodendrocyte apoptosis	Upregulation leads to elevated QUIN levels	Activation may lead to decrease in inflammation
Induces astrocyte apoptosis	Inhibition elevates KYNA levels	Inhibition can cause more severe or less severe disease course based on timing
Induces neuronal apoptosis	Inhibition leads to better cognitive performance in animals	

QUIN, quinolinic acid; KMO, kynurenine 3-monooxygenase; IDO-1, indolamine 2,3-dioxygenase; KYNA, kynurenic acid.

**Table 3:** Kynurenine pathway metabolites and enzymes—results of in vivo studies.

QUIN	KYNA
Elevated levels in the CSF of MS patients	Decreased levels in the CSF of MS patients
Elevated levels in the CSF RRMS patients to the point of being one of the best predictors of disease severity	Decreased levels in the CSF SPMS patients
Elevated levels in the CSF of PPMS patients	Elevated levels in the CSF of PPMS patients

QUIN, quinolinic acid; KYNA, kynurenic acid; CSF, cerebro-spinal fluid; RRMS, relapsing-remitting MS; SPMS, secondary progressive MS; PPMS, primary progressive MS.

## Data Availability

Not applicable.

## Abbreviations

3-HAA	3-hydroxyanthranilic acid
3-HAO	3-hydroxyanthranilate oxidase
3-HK	3-hydroxy-l-kynurenine
9HP	9-hole peg test
AA	anthranilic acid
AD	Alzheimer’s disease
AMPA	α-amino-3-hydroxy-5-methyl-4-isoxazolepropionic acid
A*β*	*β*-amyloid
BBB	blood–brain barrier
BICAMS	brief international cognitive assessment for multiple sclerosis
BVMT-R	brief visuospatial memory test
CDA	confirmed disability accumulation
CGRP	calcitonin gene-related peptide
CI	cognitive impairment
CIS	clinically isolated syndrome
CNS	central nervous system
CVLT-II	California verbal learning test
CSF	cerebrospinal fluid
DMT	disease modifying therapy
EAE	experimental autoimmune encephalomyelitis
ECL	electrochemiluminscent assay
EDSS	expanded disability status scale
ELISA	enzyme-linked immunosorbent assay
GPR35	orphan G-protein coupled receptor
HTT	huntingtin gene
IDO	indolamine 2,3-dioxygenase
IFN-*γ*	interferon-*γ*
JCV	John Cunningham virus
KAT	kynurenine aminotransferase
KMO	kynurenine 3-monooxygenase
KP	kynurenine pathway
KYNA	kynurenic acid
L-KYN	L-kynurenine
LP	lumbar puncture
mCGL	macular ganglion cell layer
MHC	major histocompatibility complex
MRI	magnetic resonance imaging
MS	multiple sclerosis
MSFC	multiple sclerosis functional composite
NAD+	nicotinamide adenine dinucleotide
NADP+	nicotinamide adenine dinucleotide phosphate
NEDA	no evidence of disease activity
NfL	neurofilament
NMDA	*N*-methyl-d-aspartate
OCT	optical coherence tomography
ON	optic neuritis
OPN	osteopontin
PASAT	paced auditory serial adding test
PD	Parkinson’s disease
PetCO_2_	end-tidal partial pressure of CO_2_
PIRA	progression independent from relapse activity
PIRMA	progression independent from relapse and MRI activity
PNS	peripheral nervous system
PPAR-*γ*	peroxisome proliferator-activated receptor-*γ*
PPMS	primary progressive multiple sclerosis
QoL	quality of life
QUIN	quinolinic acid
RA	radial artery
RGC	retinal ganglion cell
RIS	radiologically isolated syndrome
RNFL	retinal nerve fiber layer
ROS	reactive oxygen species
RRMS	relapsing-remitting multiple sclerosis
SDMT	symbol digit modalities test
SiMoA	single molecular array
SPMS	secondary progressive multiple sclerosis
STA	superficial temporal artery
T25W	timed 25-feet walk test
TDO	tryptophan 2,3-dioxygenase
TNF-α	tumor necrosis factor-α
Trp	tryptophan
VMCA	blood flow velocity of the middle cerebral artery
WBA	whole brain atrophy
WHO	World Health Organization
